# Medical News and Miscellany

**Published:** 1894-10

**Authors:** 


### Medical News and Miscellany.

**Dr. J. T. Field**, of Fort Worth, is taking a post-graduate course at Chicago.

**Erratum.**—Wrong head to Prof. Smith's Department. Discovered too late for correction.

**For Sale.**—Practice of $2,000, on railroad. Small village, good churches and schools. Will sell residence and give practice and good will. Address Dr. R. H., care TEXAS MEDICAL JOURNAL, Austin, Texas.

**Dr. B. F. Church**, First Assistant Physician at the State Lunatic Asylum, at Terrell, Texas, was married in Lynchburg, Virginia, on the 26th September ult., to Miss Mabel Stuart, daughter of Mrs. J. H. Miller, of Lynchburg.

**The American Medical Publishers Association** will hold their next meeting in Baltimore, June 5, '95, just prior to the meeting of the American Medical Association. For information address Dr. C. W. Fassett, Secretary, St. Joseph, Mo.

**The Concho Country.**—Dr. Conerly, in his paper on tuberculosis, in this issue, in enumerating the attractions of the Concho, omits to mention the fact that for bass fishing and for deer, turkey, and other game, the Concho valley is unsurpassed.

**Free Lectures.**—Dr. Frank Lydston will give a special course in Regional Surgery at the Masonic Hospital, Chicago, lecturing every Monday at 8 p. m., beginnning October 1st. The lectures will be illustrated by special charts and drawings, and are free.

**Married** at Dallas, Texas, October 3d (inst.), Dr. Samuel E. Milliken, of New York, to Miss Sallie Gibbs, daughter of Ex-Gov. Barnett Gibbs, of Dallas. Dr. T. J. Bennett, editor of *Texas Sanitarian*, officiated as *best man*. The "Red Back" was not in it; no cards and no cake.

**A cyclone** struck the insane asylum at Little Rock, Ark., on the 2d of October, (inst.), demolishing three stories of a wing containing 260 patients. Dr. Ingate, one of the medical staff, and formerly of Mobile, Ala., was killed. Two of the patients were killed also. It is most remarkable that there were not many more killed.

**Clubs.**—Doctor, don't forget that you can get the *Literary Digest* the best weekly literary publication extant, the price of which is $3, for $2.40 by clubbing with the "Red Back." Both for $4.40.

"Recollections of a Virginian," by General Dabney H. Maury, price $1.50; in club with this journal, both for $3.03. Address this office.

---

**To Kill Red Ants.**—Pour a wine-glass-full of bi-sulphide of carbon into the entrance to the nest, and apply a match, having previously placed a brick or flat stone over the opening to confine the vapor. The carbon ignites with a flash like powder, and will burn for several minutes. Be careful to not inhale the vapor. It is rarely necessary to repeat the dose; it is a "dead shot."

---

**Very Extraordinary** (if **True**).—Mr. Clark Bell, in his paper on "Railway Spine," read at the meeting of National Association of Railway Surgeons, and published in the *Texas Sanitarian* and *Galveston News*, says, speaking of railway spine: "It is not found away from railroads, and is absolutely unknown amongst savages"! The savages ought to be ashamed of themselves.

---

**Imitation the Sincerest Flattery.**—Dr. Boteler, of Kansas City, will shortly issue a new medical journal, patterned after the "Red Back," and writes us that he considers the TEXAS MEDICAL JOURNAL nearer his ideal than any he has seen, and that he will endeavor to make his journal as near like it as possible.

There is nothing like success, unless it is originality; they both captivate.

---

**Yellow fever** has knocked at our doors several times, but has been told "thus far and no farther." The maritime system of quarantine has been brought to such perfection, the officers on duty at the several ports being all experienced,—experts in yellow fever, in fact, and the knowledge of the ways and doings of this plague is such now, that it would be surprising indeed—and it could occur only by an accident or by treachery on the part of some ship captain—if the disease should ever again enter Texas.

---

**Dr. Fuller's paper** on hot intra-peritoneal injections of salt water for hemorrhage will be read with much interest. If the doctor is not the inventor of the method he deserves credit for reviving it, and giving his experience to the profession. The method will constitute a valuable addition to the physicians' resources; and its applicability is not confined alone to hemorrhage, but it would seem to be a most rational remedy in cholera, or cholera nostra. The doctor spoils the effect by the closing paragraph: he has "said more good" in the paper, than "wishing no one may ever be called on to use the method;" he has given the profession a boon, even should it be used *only* as a last resort.

---

**Mississippi Valley Medical Association.**—For the Mississippi Valley Medical Association meeting at Hot Springs, Ark., November 20th to 23d, the International Route, I. & G. N. R. R., will make rate of one fare for round trip from all points on its line to Hot Springs and return. Tickets on sale November 17th to 20th inclusive, and limited to 20 days for return.

Call on nearest ticket agent for full informattion, or address

D. J. PRICE, A. G. P. A.,
Palestine, Texas.

---

**Prof. West Married.**—On the 4th of September ult., at the Presbyterian church in Winfield, Kan., the home of the parents of the bride, Dr. Hamilton A. West, Professor of Theory and Practice of Medicine in the Medical Department of University of Texas, Galveston, was united in marriage to Mrs. Ella May Tuller, of Galveston, daughter of Capt. and Mrs. J. B. Fishback, of Winfield, Kansas. Dr. and Mrs. West will be at home in Galveston after 1st of October, instant. They took a trip through Canada, Saratoga, New York and the East generally. The JOURNAL extends congratulations to the handsome young professor. He will be a better lecturer, a better doctor, a better man, and better every way for having taken a "better half."

---

**An outrage.**—Dr. Pilcher, superintendent of the institution for imbeciles and weak minded children, at Winfield, Kan., has been bitterly denounced by newspapers in Winfield and Topeka, for castrating several boys—inmates—who are confirmed masturbators. His predecessor, Dr. Wile, had treated these boys five years without benefit, and Dr. Pilcher, taking a rational view of the subject, performed the operation for the same reason he would perform any other surgical operation—for its curative effect. There is a strong probability that he will be indicted for mayhem, to the everlasting disgrace of the civilization of the 19th century. Brockway—the monster—who practiced "moral suasion" with a paddle on the bare back of the inmates of Elmira Reformatory, goes scott free. We will have a leader on this subject in our next.

---

**No Respecter of Persons.**—The venerable President of the Board of Regents of the University of Texas, Dr. T. D. Wooten, was robbed by burglars entering his house on the night of Oct. 3rd. They got away with $80 in cash and the doctor's gold watch and chain, his cuff buttons, etc. What should be done with a person so lost to every sense of decency, respect and common sense as to rob a man whose life has been devoted to the work of humanity and the relief of suffering? We recommend—like Mrs. Hale's cook book, with reference to cooking a hare—first, catch your hare; after catching this fellow, castrate him, tie him securely and have somebody read Rider Haggard's "Nada the Lily" to him, or turn Dr. Hackenslasher loose on him with an account of his "interesting cases"; he should be *bored* to death.

---

**The Texas Medical College** (Medical Department, University of Texas), opened its session Oct. 1st with over one hundred matriculants, and from letters received by the Regents and the Dean, there is every reason to believe the class will number over two hundred by the 1st of November. The facilities for thorough instruction in every department of medicine and surgery possessed by the Texas Medical College, are unsurpassed by any college in the United States; and amongst the Faculty there are some of the hardest working and most zealous, as well as the ablest, teachers to be found anywhere. It is the determination of the Regents and Faculty to make a diploma from this College an honor, which won will be worthily worn and appreciated, and a passport to every medical institution in the world. We are proud of our College.

---

**Transactions, 1894.**—The senior editor acknowledges with thanks the courtesy at the hands of Secretary West of a beautiful morocco-bound and gilt-edge edition of the State Medical Association Transactions, with name in gold. A few copies only are bound in this handsome and expensive style; they are presented to the officers and ex-officers.

The work is a credit to the Publishing Committee, and the papers, as well as the work, are a credit to the Association. Barring a few ugly typographical errors, which it seems might have been avoided, the work is a masterpiece. The edition of 500 is bound in brown cloth, uniform with the style inaugurated by Dr. West upon his accession to office, in 1891.

The glory of the achievement is only dimmed by the fact that it bankrupted the treasury to produce it.

---

**Dr. Isadore Dyer**, of the staff of this JOURNAL, and Professor of Dermatology in Tulane University, and connected with the New Orleans Polyclinic, is off on a summer trip "for recreation," he says, but while he is resting he will, "after seeing a little dermatology, get an insight into the details of the working system and house plans of the Post Graduate Institutions of New York and Philadelphia." The N. O. Polyclinic he says, is outgrowing its present facilities and the faculty expect to build in the near future. While "resting" Dr. Dyer,—the indefatigable—will also buy the furniture for the New Orleans Sanitarium, of which institution he is Secretary. The doctor writes us that he "intends to enjoy his rest to its full.'' If he can do so while engaged at such hard work, he excels the old lady who always said she was enjoying very bad health.

He will take observations for future notes for his department in the Red back, and will be at his post in New Orleans by October 1st.

[Intended for September number, but failed to connect.—ED.]

---

**Medical Examining Board, 8th District.**—In accordance to a notice from Judge E. W. Terhune, the following named members of the newly appointed Medical Examining Board for the 8th Judicial District met in the office of Dr. E. G. Cochran, at Greenville, Texas, on September 5th, for the purpose of organizing and electing officers: Dr. A. E. Garrett, of Sulphur Springs; Dr. F. A. Ramsey, of Wolf City; Dr. C. M. Harrison, of Cooper; Dr. M. Smith, of Black Jack Grove, and Dr. E. G. Cochran, of Greenville.

Dr. Richardson, of Ben Franklin, and Dr. Duffey, of Emory, were appointed on the new board, but were not present.

Dr. A. E. Garrett was elected President, and Dr. E. G. Cochran, Socretary.

The regular meetings of the board will be the second Mondays in March and September. A meeting of the board will be held in Greenville, Texas, at the office of Dr. E. G. Cochran, on October 1st, at 2:30 p. m., at which time all applicants who come before the board will be examined.

**The State Lunatic Asylum**, at Austin, under the management of Superintendent F. S. White, is a credit to the great State of Texas, and what it was intended to be—an asylum for the incurably insane, and a hospital for the treatment of the acute cases. Dr. White belongs to the present day; he is a man of ideas and of progress. He has instituted several reforms and made much improvement in the institution during his incumbency which are a credit to him. There is, about the establishment and the grounds, an air of repose; it is certainly a very quiet insane asylum; one entering its precincts for the first time would hardly suppose it was a "mad house." The Doctor evidently appreciates the influence of environment in ministering to the mind diseased, and has made the grounds a thing of beauty and a joy. He is a strong advocate of castration as a therapeutic measure, and with the JOURNAL, would like to see the operation—at the physician's discretion—legalized, but until it is made lawful he would hardly do as Dr. Pilcher did, let his conviction of what *ought to be* the law, induce him to resort to the remedy. We hope the legislature will make such a law.

---

**The North American Medical Review** has made its appearance. Vol. 1, No. 1, is on our table. It is edited and published by Dr. Boteler, of Kansas City, and is an exact imitation of the TEXAS MEDICAL JOURNAL in paper, type and general get up, *red cover* and all; so much like it indeed that we are too modest to bestow upon it the measure of praise it really deserves—we might be thought *vain.* We will accord to it as graceful a reception as is possible in the nature of things, and to do so, must restrain a quite natural impulse:

"When she comes all dressed in *red*
Take a stick and break her head!"

The "Red Back" had not preempted a claim to red, nor had we trade-marked our cover—wish we had; still we would rather Brother Boteler had chosen some other color, the *red back* had become so characteristic of the TEXAS MEDICAL JOURNAL that we can't help feeling it is a "sorter" infringement on its rights. However, we pride ourselves more on our "insides" than we do on our "outsides," and will try to set the N. A. M. R. a pretty lively pace if it is disposed still to imitate us. Success to the "K. C. Red Back," any how.

---

**Professors Duncan Eve and Frank Glenn** announce that in consequence of differences in the Faculty of the Medical Department, University of Tennessee (Nashville Medical College), as to the business policy adopted by the Faculty, they have severed their connection with that institution, and resigned their respective chairs. They say:

"Being the originators and having labored hard for the success of the College with which we have so long been identified, we can not help but express our regrets at being compelled to withdraw.

"In the meantime, and for next winter, we hereby announce that we intend to conduct a private class in our special work, operative and clinical surgery, and genito-urinary with venereal diseases.

"Yet being in the prime of life, full of energy and hope, we take this method of informing our many friends that it is our expectation to take steps quite soon for the organization of another medical college. While we agree with the almost unanimous opinion that there are enough medical colleges in the country, we can not help indulging the thought from our experiences under very trying circumstances, that we can make a success of the prospective one we have now in view."

For information, address Dr. Eve or Dr. Glenn.

---

**The Kentucky School of Medicine.**—The following brief note, for which the *Medical Record* of New York is responsible, is calculated to mislead those who read it without investigating the real facts in the case:

"At the meeting of the Association of American Medical Colleges held in San Francisco on June 7, 1894, the Kentucky School of Medicine, of Louisville, Kentucky, was dropped from membership in the association."

The real truth of the matter is that the Kentucky School of Medicine was never a member of the American Medical College Association, but the requirements in the catalogue recently issued are higher than are the requirements of that Association. The school has been conducted in strict accordance with the requirements observed by most successful and reputable colleges, and adheres strictly to the three year course, and no school has been more respected by the honorable members of the medical profession. In laboratory, didactic and clinical work the school has adopted the most approved methods, and now that the faculty have completed a large hospital adjoining the college, no school in the country can offer better practical and clinical advantages.

The college is a member of the Southern Medical College Association, and its requirements are really higher and more rigid than those of either of the college associations.—*Medical Progress.*

**Ho! for Hot Springs!**—What a splendid opportunity will shortly be presented to go to Hot Springs, Ark., without being suspected of having to "*have* to!" and what a glorious good time is in store for ye doctors! The great Mississippi Valley Medical Association will meet there November 20 to 23d, and the preparation for a grand re-union, a feast of reason, and a flow of —well—wine—amongst other things—is on a gigantic scale. The committee of arrangements have literally put the little pot in the big one and seasoned with its legs. They have worked the railroads for half fare and the hotels for reduced rates, and oh! what a crowd will be there. Let it not be forgotten that at our April meeting of the Texas State Medical Association an invitation to attend in a body was read, and by resolution accepted; so we have just got to go. The program promises a treat. The big guns will be heard from. Dr. X. C. Scott, of Cleveland, is President (an x-c-lent fellow), and he will make things hum. If any of our readers want to know more about this mass meeting (blue mass), let them drop a postal card to Dr. T. E. Holland, the chairman of committee of arrangements, at Hot Springs, for one of those lovely little folders, descriptive of everything in and about Hot Springs. In this connection, see the advertisement of the great Missouri Pacific and leased lines route in this issue. Tickets at half rate will be on sale everywhere in Texas from November 18, good for twenty days.

---

**Pay-day.**—Bills were mailed October 1st to all our subscribers whose subscription has expired, and we hope all who can do so will promptly renew. Especially those who have, from inadvertence or neglect, permitted their subscription to get far in arrears, are called upon to remit, at least in part; and those farthest behind may expect a draft, if not heard from shortly. We earnestly hope they will be prepared to honor it. Pay day ought to come *once in awhile*, and we have waited on quite a number of our friends quite "a while." Drafts will be made on all who are over two years in arrears. We need money to keep the "Red Back" up to the top notch of excellence, and we must urge our delinquent friends, as Miss Tox did Mrs. Dombey, to "make an effort."

---

**October** is the month of cotton, calves, colds and collections. We are not interested in any of these "forces" (four c's) except the latter, and we do earnestly hope and expect that our good friends will not forget our patience and long suffering (nor their patients and their long suffering) when they go out in quest of cash, or when they sell their cotton, cows, calves, or when they collect any cash (this is the "c" c'son, see?) but will promptly divide with us.

Heaped in the hollow of his [our] desk
The doctor's [the Journal's] bills are laid.
He'll [we'll] rustle 'mongst his patrons [our subscribers] till
The last darned one is paid;
The farmer [the doctor] and his men are paid,
And in their eyes he sees [we see]
—Speculation? No; but cash,
To pay, in lieu of peas!

[*Back number revised.*]

[We do not want any peas in ours, if you p'ease.—ED.]

---

### AS OTHERS SEE US.

---

"The TEXAS MEDICAL JOURNAL is the very best journal I have seen in the West."—DR. W. C. BOTELER, Kansas City.

Dr. W. B. Outten, the well known Chief Surgeon of the great Missouri Pacific Railway Company, says: "I am always well pleased to receive the 'RED STAR of TEXAS.' It is strong, vigorous and aggressive, and to my conception—thoroughly wide awake and progressive." [That's the size of it.—ED.]

Dr. I. N. Love, whom everybody knows, says: "Let me congratulate you upon the continued attractiveness of your JOURNAL. It makes me weary to see some of the medical journals that are wafted through the mails, that are better adapted to fill the waste-basket. Doctors all over the country see a journal succeed, and they fancy they can go and do likewise. They fail to realize that it takes journalistic instinct—talent,—hard work, and the expenditure of a great deal of money to make a journal succeed, as you can no doubt well testify."

[It is related of Goldsmith that he was envious of the success of anybody, in any line, and was vain enough to say that what any one else could do, he could do. He laid a wager with Garrick that he could perform an acrobatic feat that he had seen done at a circus, and actually attempted it. He failed, of course, and got his shins skinned for his pains. We have seen some similar foolhardiness and failures in the attempt to establish a medical journal "to fill a long-felt want."—ED.]

One of the largest drug firms of the United States, a long-time patron of the Red Back, writes: "We think your JOURNAL one of the best of its kind, being so bright and original, and so widely circulated throughout Texas." [A representative of this firm, who traveled through Texas, informed us that he saw the Red Back everywhere he went.—ED.]

Prof. Geo. Dock, M. D., of University of Michigan, in renewing his subscription, writes: "Please continue to send the Red Back. I enjoy the reading about the good work going on in Texas, and hope you will keep it going."

---

### A GREAT SANITARIAN ON SPINAL CONCUSSION.

In such a duel as is presented by the contest between railway surgeons of the country, in behalf of their corporations, on the one hand, acting in self-defence, as in some cases where their very existence is threatened by the enormity of the demands made by the rapacity of claimants and their counsel, legal and medical, and by claimants represented by distinguished and able members of both professions, we must expect to see sturdy blows given and taken, and we must look for the strongest and ablest presentation of the side of the claimant, who will not consent to surrender without a struggle, and a strong one at that.

The publication in the *Texas Medical Journal* and the *Galveston News* of the paper entitled "Railway Spine," read by the editor of this journal before the National Association of Railway Surgeons at the Galveston meeting last May, and an article by Dr. D. R. Wallace, the veteran surgeon of Waco, Texas, in the TEXAS SANITARIAN, advocating similar views, has drawn out the State Health Officer of the great State of Texas, Dr. R. M. Swearingen, of Austin, a capital good fellow, and a physician of high character and standing in his State, who assails the authors of the papers, and the views presented with great vehemence. He regards the great body of railway surgeons as under the domination of the chief surgeons of their respective roads.

I quote his language, speaking of the more prominent of the chief surgeons of the National Association of Railway Surgeons:

"There are others, we think, who are inspired by less praiseworthy objects, and in this class will be found some of the strongest men among them. They are the 'chiefs (not all, however) of the hospital departments' for great railway systems. These are the men who formulate rules for the guidance of subordinates, furnish the schedule of charges to be made for the services rendered by them to employes, pay them their pitiful fees, mould their opinions in harmony with the new dispensation, teach them how to be experts in all manner of railway injuries, particularly the railway spine, and, above all things, to store their minds with useful knowledge, when called upon to give testimony in suits for damages.

"I do not mean to convey the impression that the subordinate surgeons thus taught and drilled will ever intentionally bear false witness, or in any manner do violence to the most stainless conscience. The object of that corporation school of surgery is to organize a corps of doctors who think alike; who believe Gross and Erichsen teach dangerous and mischievous doctrines; and who are always ready for duty when called upon to give pointers to attorneys, or give evidence in courts of justice.

"Dr. W. B. Outten, of St. Louis, one of the chiefs, and an ex-president, I think, of the Association, occupies a high seat in this modern school of surgery, and might be designated as the Professor of "Theory and Practice." In the *Galveston News* of May 11th, he gives some racy theories on the causes of railway concussions."

Dr. W. B. Outten, chief surgeon of the Missouri Pacific railway system, especially falls under his malediction, as do other prominent gentlemen.

I do not believe that Dr. Swearingen, one of the good fellows of the Medico-Legal Society, has in the past established a great reputation as a medical witness in Texas, in railway damage cases. If he has done so, it has escaped my observation. Nor do I think his motive is to bring forward his name as a future aspirant in the courts of Texas, in favor of mulcting improperly and exorbitantly the railways of a State which owes so much to railways for its wonderful growth, progress, and development as Texas does.

Dr. Swearingen is one of the ablest of the sanitarians of the South and West. It is due to his magnificent personality that he has created for the State of the Lone Star a sanitary system as unique as it is excellent. On that he is high authority. It does not disparage his high character as a sanitarian to say that he is not regarded as an authority in damage cases.

Will he oblige the surgeons of the country, and the bench and the bar, with a definition of "concussion of the spine," so that the average juryman or judge will be able to recognize it when he sees it? What has he to say of the criticisms upon Erichsen's definition in the article he assails? What of the opinion of Dr. Joseph Jones, of New Orleans, there quoted? Is there such a thing, Dr. Swearingen, as "concusssion of the spine uncomplicated by external lesion of the vertebral column?"

We must be more cautious of impugning the motive of railway surgeons or of railway counsel.

Accidents on railway trains sometimes fracture and break the spinal column. The case cited of a fractured or broken spine of the section hand injured near Manor, Texas, in charge of Dr. Gregg, is one of the rare cases of this kind.

No one can defend such an order, in such a case, as was given, but the case and the order are quite foreign to the discussion of cases of "concussion of the spine uncomplicated by external lesion of the vertebral column."

Should any steps be taken by honorable surgeons towards arresting the recognized evil of fraudulent claims, constantly presented against railways, under the class designated as railway spine or spinal concussion?

It is fortunate for the new Section on Railway Surgery of the Medico-Legal Society that it is as open to the surgeon of the railway corporation as to the attorneys or surgeons of the claimant.

More of the surgeons of the Medico-Legal Society are probably not surgeons of railways than those who are. And this is true of the legal side also. Is it not time to provide remedies for acknowledged evils, before they become unbearable?

I am obliged to the great Texas sanitarian for his assault, if it will serve to open discussion on an evil so momentous and important as railway spine has grown to be.

Dr. Swearingen's criticisms upon railway surgeons, their intelligence, honor, and motives, are without question ill-timed, not well considered, and should be withdrawn.

It is a question higher than the personal motives of men or of individuals. The evil is colossal, universally recognized, and a scandal and disgrace of our civilization. How to remedy it is well worthy the serious and thoughtful inquiry of every honorable surgeon in the land.—*N. Y. Medico-Legal Journal* (advance sheets), September, 1894, Clark Bell, Esq., editor.

